# Level of difficulty of tooth extractions among roughly 100,000 procedures in primary care

**DOI:** 10.1007/s00784-023-05073-4

**Published:** 2023-05-26

**Authors:** Oona Lindahl, Irja Ventä

**Affiliations:** Department of Oral and Maxillofacial Diseases, Faculty of Medicine, University of Helsinki, P.O. Box 41, 00014 Helsinki, Finland

**Keywords:** Tooth extraction, Primary healthcare, Decision making, Oral surgical procedures, Dental care, Dental caries

## Abstract

**Objectives:**

The study examined treatment codes of extracted teeth and aimed to assess degree of difficulty concerning all tooth extractions.

**Materials and methods:**

Retrospective data on treatment codes of all tooth extractions during a two-year period were obtained from the patient register in primary oral healthcare of the City of Helsinki, Finland. Prevalence, indication, and method of extraction appeared in the treatment codes (EBA-codes). Degree of difficulty was determined from the method and classified as non-operative or operative and as routine or demanding. Statistics included frequencies, percentages, and χ^2^ test.

**Results:**

Total number of extraction procedures was 97,276, including 121,342 extracted teeth. The most frequent procedure was a routine extraction of a tooth with forceps (55%, *n* = 53,642). The main reason for extraction was caries (27%, *n* = 20,889). Of the extractions, 79% (*n* = 76,435) were non-operative, 13% (*n* = 12,819) operative, and 8% (*n* = 8,022) multiple extractions in one visit. Level of difficulty was distributed as routine non-operative (63%), demanding non-operative (15%), routine operative (12%), demanding operative (2%), and multiple extractions (8%).

**Conclusions:**

Two-thirds of all tooth extractions in primary care were relatively simple. However, 29% of procedures were classified as demanding.

**Clinical relevance:**

As earlier methods for assessing level of difficulty were aimed at third molars alone, an analysis was presented for all tooth extractions. This approach may be useful for research purposes, and the profile of tooth extractions and their difficulty level may be practical also for decision-makers in primary care.

## Introduction

Extracting a tooth may be a simple procedure with forceps or a demanding operative extraction of a deeply impacted tooth with a cyst. Several methods have been developed for assessing the level of difficulty of extractions; however, these methods are aimed at third molars alone. The most popular and long-term methods are based on inclination of the tooth and depth in the bone [1, 2]. Recently, deep learning models have also been applied to these two variables [3]. In addition to these radiographic assessments, the most recent methods include also clinical and demographic variables [4, 5], which are evaluated to be significant factors affecting surgical difficulty of third molar extractions [6]. As regards the extraction of teeth other than third molars, the inclination is mostly vertical and there is usually no variation in depth in the bone. However, the degree of difficulty of extracting teeth other than third molars has been rarely (if ever) examined. Obviously, the degree of difficulty is clinically assessed before every tooth extraction; nevertheless, to our knowledge, no studies have been published for teeth other than third molars.

The literature offers an abundance of studies on tooth extraction at general dental practices, with topics such as prevalence of extraction, reason for extraction, age of patients, and distribution of extracted teeth [7–11]. Data for these studies were collected either by nationwide questionnaires sent to dentists [7–9, 12–14] or by retrospective analysis of patient records from one unit [10, 11, 15]. To evaluate the level of difficulty of extractions, it seems that the retrospective method would be an appropriate approach to analyze actual completed treatments [16]. However, the level of difficulty of extractions was not reported in any of the previous studies.

The main reasons for extracting teeth are caries and periodontal diseases [7–14, 17, 18]. Among patients aged less than 20 years, the reason for tooth extraction is relatively often orthodontic [8, 9, 12, 14, 17, 18]. Caries predominates up to middle age, followed by periodontal reasons at later ages [11, 14]. Peak age of tooth extractions of all teeth among 15- to 81-year-old subjects at a university dental clinic is 45 to 64 years [11]. Third molars are extracted from relatively young subjects, mostly aged between 20 and 30 years [8], and at a peak age of 23 to 25 years [19]. Young people have mostly premolars and molars extracted, and older people incisors and canines [10, 11].

In the literature, the prevalence, indications, and patient characteristics at tooth extraction are well documented. However, the method of extraction, and consequently, the level of difficulty of extractions have not been investigated, except for third molars. The aim of this study was to examine treatment codes of extracted teeth in primary care and to assess the degree of difficulty concerning the extraction of all teeth. Our further aim was to uncover new information on tooth extractions that would be useful for clinical or research purposes.

## Materials and methods

A retrospective study was designed on statistics drawn from the electronic patient register from the Department of Social Services and Health Care of the City of Helsinki, Finland. During the study period, the population of Helsinki comprised approximately 613,000 inhabitants [20], all of whom were eligible to use the services. In addition to scheduled appointments, the services included acute dental care provided at daytime, in the evenings, and on weekends. The acute care covered the inhabitants of the five neighboring cities (Helsinki, Espoo, Vantaa, Kauniainen, and Kirkkonummi), with the total catchment area having 1.1 million inhabitants in 2013 [20]. The services did not include extractions performed at hospitals or in private clinics. During the study period, the staff of the healthcare unit consisted of approximately 200 dentists, ten of whom were oral and maxillofacial surgeons. The studied unit, the Social Services and Health Care of the City of Helsinki, is located in the capital of the country and is the largest unit nationwide providing public services.

From the patient register, statistics were acquired of treatment codes of all tooth extractions carried out over a nearly two-year period from 1 January 2013 to 8 December 2014 (100 weeks). The extractions included also deciduous teeth and third molars. Extractions in this healthcare unit are performed under local anesthesia in 93% of cases [19]. The treatment codes of all extraction procedures, beginning with letters EBA, had been recorded in digital patient documents by the attending dentists by his/her judgement immediately after the procedure was finished. The classification of the procedures by treatment code is a Finnish version of the Nordic Medico-Statistical Committee’s (NOMESCO) Classification of Surgical Procedures [21]. The Finnish version is published on the internet by the Finnish Institute for Health and Welfare [22], is updated annually, and is used nationwide. A treatment code describes the technical extraction procedure (Table 1). For example, EBA00 is an extraction executed with elevator and forceps, whereas EBA05 is a demanding extraction that usually means separation of a tooth without raising a flap.

**Table 1:** Classification of treatment codes for tooth extractions based on the Finnish Institute for Health and Welfare [22]

Code	Operation	Definition
EBA00	Extraction	With elevator and forceps
EBA05	Demanding extraction	Separation of tooth without raising a flap
EBA10	Operative extraction	Raising a flap, followed mostly by osteotomia and separation of tooth
EBA12	Demanding operative extraction	Deep and difficult impactions
EBA15	Extraction of several teeth	At least four teeth from a jaw at an appointment
EBA20	Hemisection	Raising a flap, separation, and extraction of a root
EBA30	Extraction of root	With elevator and forceps
EBA40	Apicoectomy, single-rooted	Apical surgery
EBA45	Apicoectomy, multi-rooted	Apical surgery
EBA99	Other operative extraction	E.g. partial extraction (coronectomy)

The study variables analyzed from the treatment codes were the number of extracted teeth, indication for extraction, and method of extraction. The number of extracted teeth was the number of corresponding extraction codes. An exception was the code EBA15, which denotes to extraction of at least four teeth per jaw in a visit, often as part of infection control. Therefore, the number of this code was multiplied by four to get an approximate value for the number of teeth extracted.

Indication for extraction was assessed from the modified classification, in which the indication for extraction was defined with a suffix letter (Table 2). The suffix was attached to three treatment codes: EBA00 (routine non-operative), EBA05 (demanding non-operative), and EBA15 (multiple extractions). The code EBA15 can include various indications for separate teeth, yet only the leading indication was recorded in the patient register. Treatment codes for operative extractions were not associated with a suffix for indication, but the diagnosis was recorded according to the International Classification of Diseases, 10^th^ revision (ICD-10) [23] on patient documents, which were not available to us.

**Table 2:** Detailed classification for indications of tooth extractions (codes EBA00, EBA05, and EBA15), with a suffix added to the end of the national code (e.g., EBA00A)

Treatment code	Suffix	Indication for extraction
EBA00, EBA05, EBA15	A	Caries
EBA00	B	Deciduous tooth
EBA00	C	Deciduous tooth (permanent missing)
EBA00, EBA05	D	Orthodontics
EBA00, EBA05, EBA15	E	Periapical periodontitis
EBA00, EBA05, EBA15	F	Periodontitis
EBA00, EBA05	G	Trauma
EBA00, EBA05	H	Other

**Fig. 1:**
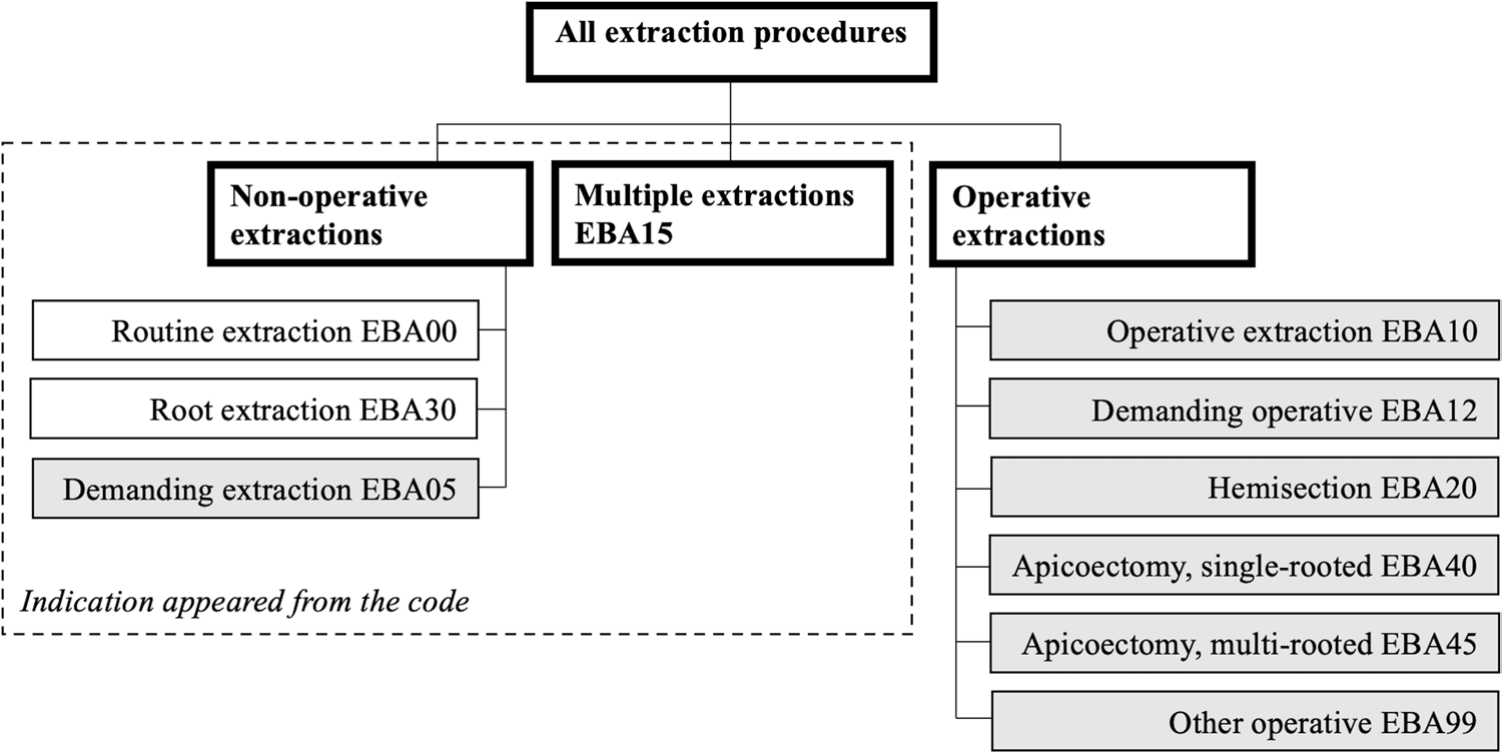
Diagram of classification of treatment codes according to indication, method, and level of difficulty of extractions. Gray boxes denote demanding extractions

The outcome variable was the level of difficulty, which was assessed from the method of extraction and was first classified into three groups: non-operative, operative, and multiple extractions (Fig. 1). Single tooth extractions were then dichotomized as routine or demanding procedures.

As several extractions were performed by the code EBA15, extractions could be non-operative or possibly also operative or a combination of these, and therefore, was presented in the analyses as its own group. Descriptive statistics included frequencies and percentages. Differences in frequencies between subgroups were evaluated using χ^2^ test. Excel spreadsheet program (Microsoft Corp., Redmond, WA, USA) was used in the analyses.

Our retrospective material did not include personal data of patients, as it was based on statistics alone. Therefore, ethics approval was not required according to the Finnish legislation. Permission for conducting this study was obtained from Social Services and Health Care of the City of Helsinki (registration no. HEL 2014–012907).

## Results

**Fig. 2:**
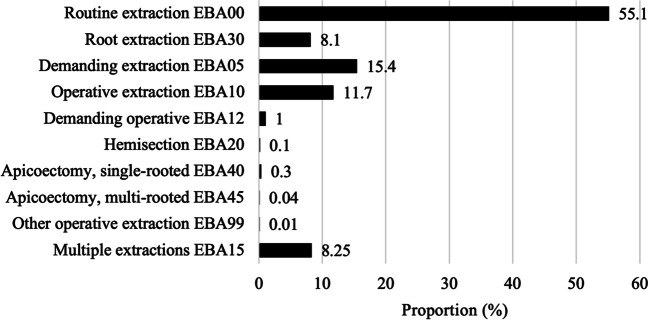
Distribution of all extraction procedures (%) according to treatment code (*N* = 97,276 procedures)

Over the two-year period, a total of 97,276 extraction procedures were performed, yielding a daily average of 139 procedures (single and multiple extractions). The total number of extracted teeth was at least 121,342, consisting of 89,254 single-tooth interventions (92%) and 8022 extraction procedures of at least four teeth in an appointment (8%), recorded by the code EBA15. Of all extraction procedures, slightly more than half were routine non-operative tooth extractions (EBA00), followed by demanding non-operative extractions (EBA05) and routine operative extractions (EBA10) (Fig. 2).

### Indications for extractions

**Fig. 3:**
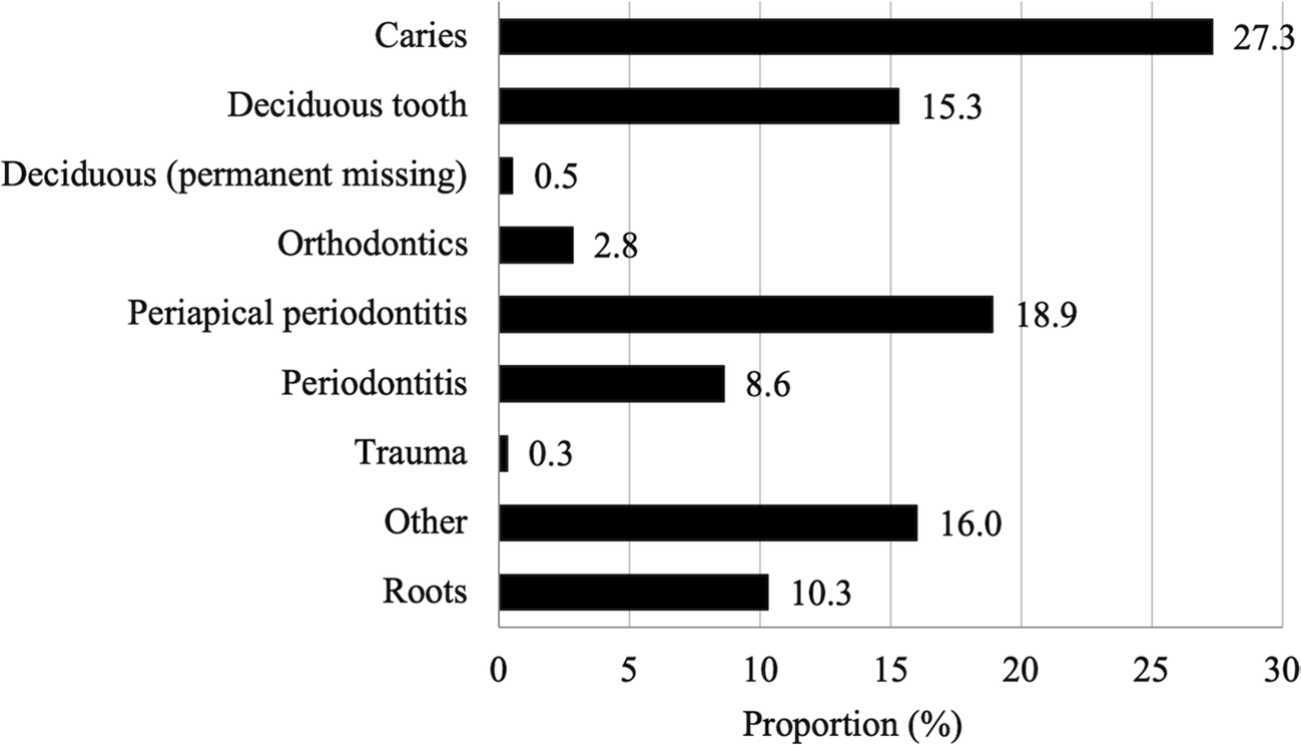
Distribution of indications for non-operative extractions (*n* = 76,435 procedures) from the codes EBA00, EBA05, and EBA30 (root)

An indication for extraction was revealed by the treatment code in 85% (*n* = 83,030) of the total number of procedures, including all non-operative extractions (EBA00, EBA05, and EBA30) and most of the multiple extractions (EBA15). Among the non-operative extractions (*n* = 76,435), the most common indication was caries, followed by periapical periodontitis and deciduous tooth (Fig. 3).

As regards EBA15 procedures, the indication was not inbuilt as a suffix in 18% (*n* = 1,427) of the procedures. For the remaining 82% of the group of multiple extractions (*n* = 6,595), the most common indication was caries (67%, *n* = 4,437), followed by periodontitis (20%, *n* = 1,315) and periapical periodontitis (13%, *n* = 843).

### Method of extractions

**Fig. 4:**
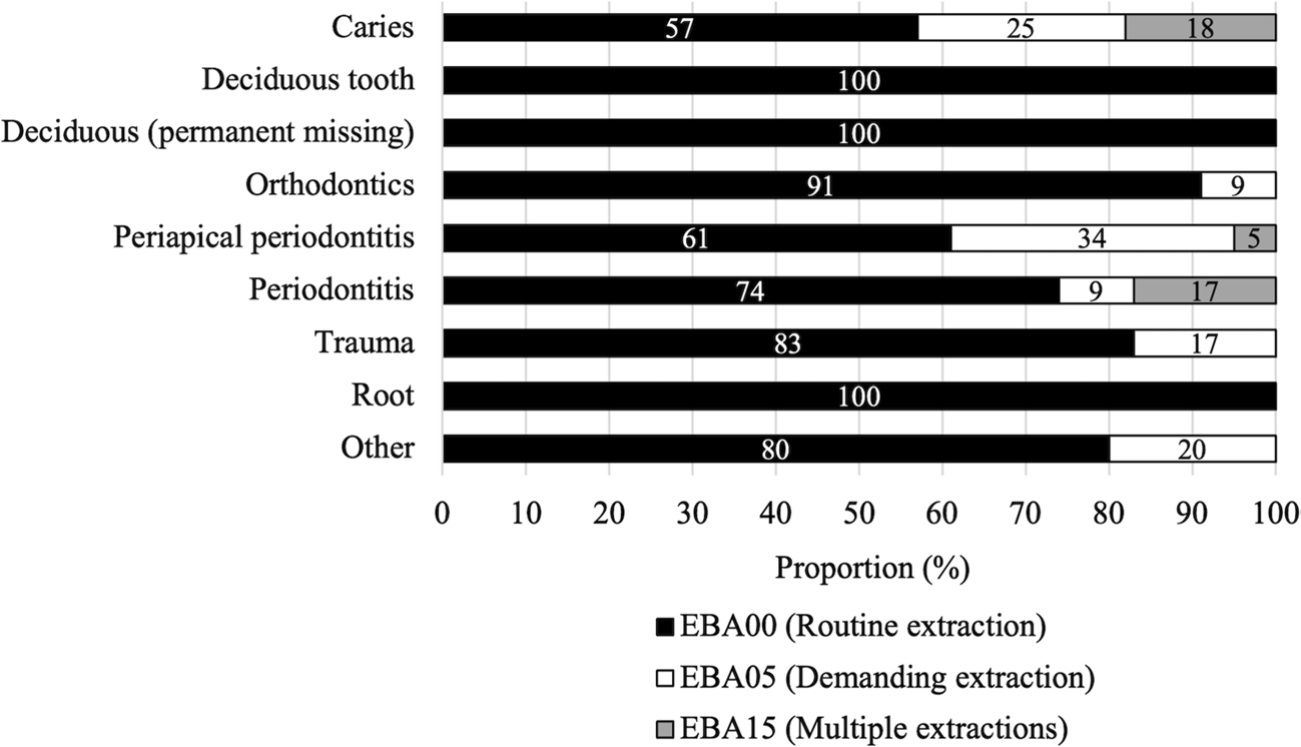
Methods of extraction distributed according to diagnoses of extraction (*n* = 83,030 procedures with diagnosis)

The methods of extraction were distributed as follows: 79% (*n* = 76,435) were non-operative extractions, 13% (*n* = 12,819) operative extractions, and 8% (*n* = 8,022) extraction procedures of at least four teeth at the same appointment. Method of extraction varied depending on the diagnosis set for the tooth being extracted (Fig. 4). For example, all extractions of roots and deciduous teeth were routine non-operative extractions with forceps. When the extraction methods and the three most common indications (caries, periodontitis, and periapical periodontitis) were cross-tabulated, teeth with periodontitis were noted to infrequently require a demanding extraction (9%, *n* = 723), while teeth with periapical periodontitis more often required a demanding extraction (34%, *n* = 5,170) (χ^2^ = 2611.78; df = 4; *p* < 0.001).

### Degree of difficulty of extractions

**Fig. 5:**
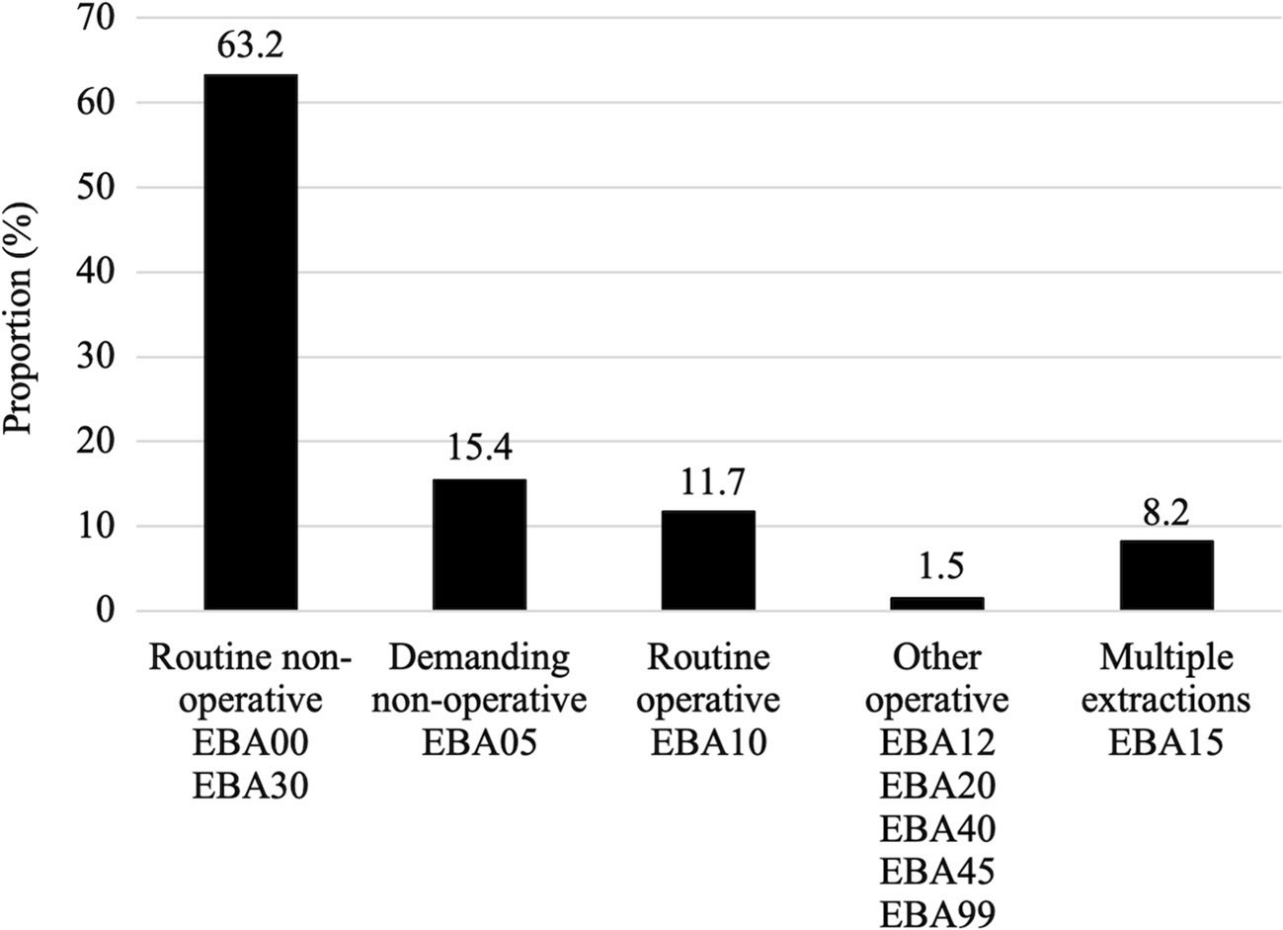
Distribution of degree of difficulty of all extraction procedures (*N* = 97,276)

Degree of difficulty of extractions could be assessed for 92% (*n* = 89,254) of the procedures, and the remaining 8% were multiple extractions. Routine non-operative extractions performed with forceps prevailed at 63% (*n* = 61,502). Various demanding procedures, including demanding non-operative extractions and all operative extractions, comprised 29% (*n* = 27,752) of all procedures (Fig. 5).

## Discussion

The purpose of our study was to examine treatment codes of extracted teeth in primary care and to assess the level of difficulty concerning extraction of all teeth. Based on our vast material of extracted teeth, the main finding was that two-thirds of the procedures were routine extractions with forceps and one-third various demanding extractions, including operative extractions. The EBA treatment codes provided a simple approach to assess the extraction difficulty for all teeth.

The coding system for tooth extractions, beginning with letters EBA, is used nationwide in all public and private healthcare units in Finland. We used the treatment codes to assess the degree of difficulty of extractions of all teeth which is a new research approach and has not been utilized previously to study this subject. Earlier studies on extractions have not evaluated the method or the degree of difficulty, except for third molars, and thus, no classification for the degree of difficulty of extractions among all teeth is available. The present findings offer a general view of extractions of all teeth and a description of proportions of different non-operative or operative extractions. Our analysis and earlier methods for the third molar differ in the approach; our perspective is postoperative, while for the third molar, it is preoperative. Thus, our way of analysis is especially suitable for research purposes in, for instance, epidemiology. The profile of tooth extractions and their difficulty level may be useful information also for decision-makers in primary care.

The usage of treatment codes is instructed by the Finnish Institute for Health and Welfare. The classification of codes includes relatively short descriptions for extraction procedures. For instance, demanding non-operative extraction code, EBA05, is defined as separation of tooth without raising a flap. Naturally, the code includes also other complicated extractions but not operative. In the end, it is up to the clinician to decide in which category the executed treatment should be recorded. However, several studies on health economics indicate that selection of treatment code or deciding on treatment includes financial incentives [24–26]. The costs of tooth extractions in private and public sector are compared in an earlier Finnish study, where it is shown that private dentists classified their tooth extractions more often as demanding (37%) in comparison to public dentists (15%) [24]. The proportion of demanding extractions in our study (EBA05; 15%) was the same as in that earlier study from Finland. Public sector dentists in Finland have fixed salaries with minor wage incentives. At the time of our data, the wage incentives were approximately €2 for EBA00 and €8 for EBA05. Having said that financial incentives matter, productivity bonuses might have affected the usage of treatment codes to some extent. Usually, private dentists in Finland work solely on fee-for-services basis. As our data did not include extractions performed in private clinics, where financial incentives seem to be more important, we expect that financial incentives had a relatively minor role in our study.

In our study, the degree of difficulty was stated unambiguously for 92% of the extractions. A surprising finding was the small proportion of operative extractions (13%), including a minimal number of specified operative extractions such as deep and difficult impactions. In another Finnish study from primary oral healthcare covering the same years as our material, the focus was on extraction of third molars alone, and that study reported that 28% of third molar extractions were operative and 72% non-operative [19]. In a US study on third molar extractions at an oral and maxillofacial unit, the proportions were reversed, with the vast majority (76%) of third molar extractions being operative (24% non-operative) [27]. Nonetheless, third molar extractions are more often operative than extractions of other teeth.

The degree of difficulty of third molar extractions has been extensively investigated for decades [1–6]. However, we failed to find earlier studies on the degree of difficulty concerning extraction of all teeth. In 2020, a systematic review of surgical difficulty in the extraction of third molars concluded that patient-specific, radiological, and operative factors were linked to demanding surgical extractions [6]. These factors comprised old age, overweight, deep impaction, unfavorable angulation or root morphology, close relation to mandibular canal or maxillary sinus, surgeon’s experience level, and complex surgical technique. The indications of extraction were not analyzed in that review. However, we examined whether the indication itself was associated with a simple or demanding extraction. The finding was obvious; teeth with periodontitis were easy to extract routinely, while periapical periodontitis rendered the extraction more demanding. Nevertheless, our analysis on the degree of difficulty would have been more thorough had it included the above-mentioned patient-specific factors. However, our way of analyzing treatment codes offers the advantage of being easy to use.

Earlier studies on extraction of all teeth between 1996 and 2020 cover countries from Europe, North America, South America, Asia, and Africa [7–15, 17, 28]. In addition to indications, other common topics in these studies have been tooth type [8, 10–12, 17], patient’s age [9–15, 17], and gender [10–15, 17]. No information on prevalence of different treatment codes, and thereby, methods of extractions or degree of difficulty was found in these studies.

Relative to earlier studies on extraction of all teeth, our material with more than 120,000 extracted teeth was exceptionally large. In previous reports, the numbers of extracted teeth vary from 554 to 11,149 [7–15, 17, 18]. Compared with our two-year study period, the corresponding period in many earlier articles is shorter, varying from one week to one month [7–9, 12–14]. Further, in most earlier studies, the data were collected using questionnaires distributed to general dentists [7–9, 12, 14], while our material comprised statistics from patient records of extractions performed by both general and specialized dentists. Similar to our protocol, a retrospective analysis of patient records from one unit was performed in studies from Italy, Greece, and Brazil [10, 11, 15]. In our study, the oral healthcare services of the City of Helsinki is the largest public services unit in the country. Most of the inhabitants younger than 18 years and almost half of the adult population in the city utilize these services [29]. Therefore, our findings describe extractions of all teeth from patients of all ages.

Most of our indication classes were the same as in many previous studies [7, 8, 11, 12, 18]. Unlike earlier studies, our indications included periapical periodontitis, deciduous tooth, and roots. Obviously, diagnosis of root remnants belongs in most cases under the heading of caries. Deciduous tooth was a peculiar indication for an extraction. However, the dental healthcare had considered it important to present this aspect separately in the statistics. Indication classes used in some earlier studies, but not in our study, were pericoronitis [7, 8, 12], (pre)prosthetic reason [7, 8, 10–12, 15, 17, 18], or impaction [12, 17, 18]. Pericoronitis and impaction are frequently associated with operative extractions; these indications were not available in our data. Differences in methods of collecting data and including indication groups make comparisons with earlier studies difficult. However, for the last 40 years, caries has been the most common reason for non-operative extractions in Finland [18].

Our findings on the degree of difficulty of tooth extractions can be generalized to healthcare units where a coding system for extractions is in use. As expected, the presently used coding system, the Finnish version of the NOMESCO classification [21] which was originally prepared for nordic countries (Finland, Sweden, Norway, Denmark, and Iceland), is not in general use. Instead, other coding systems are employed, as, for example, in the UK, codes F091 and F093 for extraction of third molars in hospitals [30]. Further research is needed on the level of difficulty of extractions in other countries. In a recent study on third molar extractions, deep learning models are conquering the field as a tool to reveal preoperatively the level of difficulty [3]. The deep learning model could potentially also be utilized to predict the degree of difficulty for all tooth extractions. The postoperative treatment codes could be linked to preoperative radiographs and to patient-specific factors to develop a deep learning model that predicts the method, and thereby, the degree of extraction difficulty concerning all teeth.

Our study had some inherent limitations due to its retrospective design. The data did not include information on patient characteristics, such as age, gender, general health status, medication, tooth type, and possible earlier endodontic treatments, all of which affect the degree of difficulty of extractions. For instance, an earlier endodontic treatment can predict a more demanding extraction or incisors with one root are simpler to extract than molar teeth with several roots. However, our treatment codes revealed exclusively whether the extraction had been non-operative or operative and whether it had been routine or demanding. Another limitation was that the indications for operative extractions were not included in the data. However, indications of extraction were not the focus of our study. A third limitation was the variety within the treatment code EBA15, representing extraction of at least four teeth in the same appointment. It was a complex code that hid information on method and indication of extractions, and therefore, we analyzed it separately. Empirically, it is known that the EBA15 code is mainly used when several totally damaged teeth are extracted in a visit with elevator and forceps to eliminate infection. Thus, the proportion of EBA15 procedures could be counted in routine extractions, as well.

With the exceptionally large material, ours is the first study to report the level of difficulty concerning extraction of all teeth, including also wisdom teeth and deciduous teeth. To classify the degree of difficulty, we used treatment codes, an approach that has not been employed for research purposes previously. For a clinician, our study provides a general view of all extractions and proportions of non-operative and operative extractions in primary care. We found that two-thirds of extractions were easy, routine extractions suitable for general dentists, and one-third were demanding. Our approach to classify extraction of all teeth according to the level of difficulty may be useful for research purposes and in decision-making in primary care.
